# A Mouse Model of Chronic West Nile Virus Disease

**DOI:** 10.1371/journal.ppat.1005996

**Published:** 2016-11-02

**Authors:** Jessica B. Graham, Jessica L. Swarts, Courtney Wilkins, Sunil Thomas, Richard Green, Aimee Sekine, Kathleen M. Voss, Renee C. Ireton, Michael Mooney, Gabrielle Choonoo, Darla R. Miller, Piper M. Treuting, Fernando Pardo Manuel de Villena, Martin T. Ferris, Shannon McWeeney, Michael Gale, Jennifer M. Lund

**Affiliations:** 1 Vaccine and Infectious Disease Division, Fred Hutchinson Cancer Research Center, Seattle, Washington, United States of America; 2 Center for Innate Immunity and Immune Disease, Department of Immunology, University of Washington School of Medicine, Seattle, Washington, United States of America; 3 Division of Bioinformatics & Computational Biology, Department of Medical Informatics & Clinical Epidemiology, Oregon Health & Science University, Portland, Oregon, United States of America; 4 OHSU Knight Cancer Center Institute, Oregon Health & Science University, Portland, Oregon, United States of America; 5 Lineberger Comprehensive Cancer Center, Department of Genetics, University of North Carolina at Chapel Hill, Chapel Hill, North Carolina, United States of America; 6 Department of Comparative Medicine, University of Washington, Seattle, Washington, United States of America; 7 Oregon Clinical and Translational Research Institute, Oregon Health & Science University, Portland, Oregon, United States of America; 8 Department of Global Health, University of Washington, Seattle, Washington, United States of America; Purdue University, UNITED STATES

## Abstract

Infection with West Nile virus (WNV) leads to a range of disease outcomes, including chronic infection, though lack of a robust mouse model of chronic WNV infection has precluded identification of the immune events contributing to persistent infection. Using the Collaborative Cross, a population of recombinant inbred mouse strains with high levels of standing genetic variation, we have identified a mouse model of persistent WNV disease, with persistence of viral loads within the brain. Compared to lines exhibiting no disease or marked disease, the F1 cross CC(032x013)F1 displays a strong immunoregulatory signature upon infection that correlates with restraint of the WNV-directed cytolytic response. We hypothesize that this regulatory T cell response sufficiently restrains the immune response such that a chronic infection can be maintained in the CNS. Use of this new mouse model of chronic neuroinvasive virus will be critical in developing improved strategies to prevent prolonged disease in humans.

## Author Summary

Much experimental work has been directed at understanding the host immune response to flavivirus infections such as West Nile virus (WNV) using mouse models of infection. While valuable information has certainly been secured using these methods, a limitation to studies with traditional inbred mouse models is that they lack the genetic diversity of the human population, and consequently this limits our ability to study particular states of disease that aren’t replicated in traditional inbred mice. For instance, despite reports of chronic WNV infection in humans, a mouse model of chronic infection is lacking. Here, we report the discovery of a mouse model of chronic WNV infection using the Collaborative Cross, a panel of reproducible recombinant inbred mouse strains with a high degree of standing genetic variation. Further, we find that chronic disease is associated with altered innate immunity and increased regulatory T cell frequency, and we find that chronically infected mice have lower expression of genes associated with cytolysis. Thus, in addition to discovery of a new chronic infection model that may be useful for other neuroinvasive infections, we provide an immunologic mechanism by which chronic virus infection can be established.

## Introduction

West Nile virus (WNV) is an emerging flavivirus, and a potential model pathogen for other viruses in its genus such as Zika, dengue, and yellow fever viruses. Since its introduction to North America in 1999, West Nile virus has spread throughout the USA to now cause significant morbidity and mortality in the Western hemisphere as well as worldwide. It is predicted that WNV and other flaviviruses will continue to cause a significant disease burden to the global population, with expansion possible particularly as mosquito habitats expand and bird migration patterns change with continued shifts in climate worldwide. WNV is neuroinvasive and can cause disease ranging from self-limiting febrile illness to disease of the central nervous system (CNS), including meningitis and encephalitis [1]. Neuroinvasive infection and CNS disease can be particularly deadly and also leave survivors with long-term physical and cognitive disabilities [2]. Additionally, a more chronic poliomyelitis-like syndrome can occur, in which patients experience neurologic weakness and/or tremor up to one year after their acute illness [2], and virus has been detected in patient urine years after the initial infection [3]. However, there is limited knowledge available regarding the immune response to WNV in humans due to the high prevalence of subclinical infection that precludes identification of WNV-infected individuals and subsequent clinical and immune response evaluations. Thus, most knowledge of anti-WNV immunity comes from study of WNV infection using inbred mouse models of infection, generally using C57BL/6J (B6) mice.

As compared to the diverse breadth of clinical outcomes and immune responses observed in the human population, the B6 mouse model of WNV infection is much less variable. When infected subcutaneously in the footpad with the contemporary emerging and neuroinvasive strain of WNV, approximately 30% of infected mice succumb to infection within 10–12 days post-infection (p.i.) p.i., depending on the inoculum dose [4–6]. While this outcome captures the processes of neuroinvasive disease of the CNS observed in human West Nile Neuroinvasive Disease, B6 mice generally display extremely similar immunophenotypes with a replicable fraction of animals transitioning to symptomatic infection. Studies in this traditional B6 model have shown that innate and adaptive immune responses orchestrate protection and control of WNV infection and disease [7]. In particular, RLR-mediated innate immunity and IFN actions are essential for restricting WNV replication and neuroinvasion to the CNS [4,6,8–11], while T cells are critical for viral clearance in the CNS. However, the *in vivo* dynamics of these innate and adaptive immune responses that associate with differential outcomes of WNV infection, and particularly a chronic infection, has not been effectively investigated and remain poorly understood.

Given the breadth in symptoms and disease severity seen in human WNV infection that are not effectively captured in the B6 model, we turned to the newly developed Collaborative Cross (CC) recombinant inbred (RI) panel to better model WNV infection and disease and understand the role of host genetics in the immune response to and control of WNV infection and disease. The CC RIs are derived from eight founder mouse strains: five classical inbred strains and three wild-derived strains [12–14]. The founder strains represent the three major *Mus musculus* subspecies, and capture nearly 90% of common genetic variation in *Mus musculus* strains (>40 million SNPs, >4 million small insertion/deletions), with this variation randomly distributed across the genome [15,16]. RI strains were created by three generations of funnel breeding to incorporate all founder strains, followed by at least 20 generations of inbreeding to develop each CC RI strain [13]. In this study, we used F1 male progeny from crosses between CC RI strains (called recombinant intercross, or RIX lines). These RIX were heterozygous for the H-2^b^ MHC haplotype, and were created by crossing two independent RI strains, one of which had the H-2^b^ allele in order to allow for the detection of WNV-specific T cells using MHC class I tetramer.

We have previously demonstrated that the CC can be used as a resource to model WNV infection in humans such that infection and comparison of CC mouse lines improves upon inbred mouse models to better capture the diversity of WNV infection outcomes observed across the genetically diverse human population [17]. Here, we further characterize the RIX line CC032/GeniUnc crossed to CC013/GeniUnc (hereafter CC(032x013)F1)), which serves as a mouse model of chronic WNV infection. While studies using B6 mice have shown that WNV RNA can persist in the CNS up to 3 months post infection in a limited fraction of mice [18], to date there is a lack of a robust mouse model of chronic WNV infection that can be used to elucidate the immune responses associated with this viral persistence and chronicity of symptoms described in human patients. Here, we characterize this RIX line compared with other RIX lines showing either no disease symptoms or significant disease, and suggest a mechanism by which WNV infection can become chronic through alterations in immune responses.

## Results

### CC(032x013)F1 hybrid mice have a unique course of disease compared to other RIX lines

**Fig 1:**
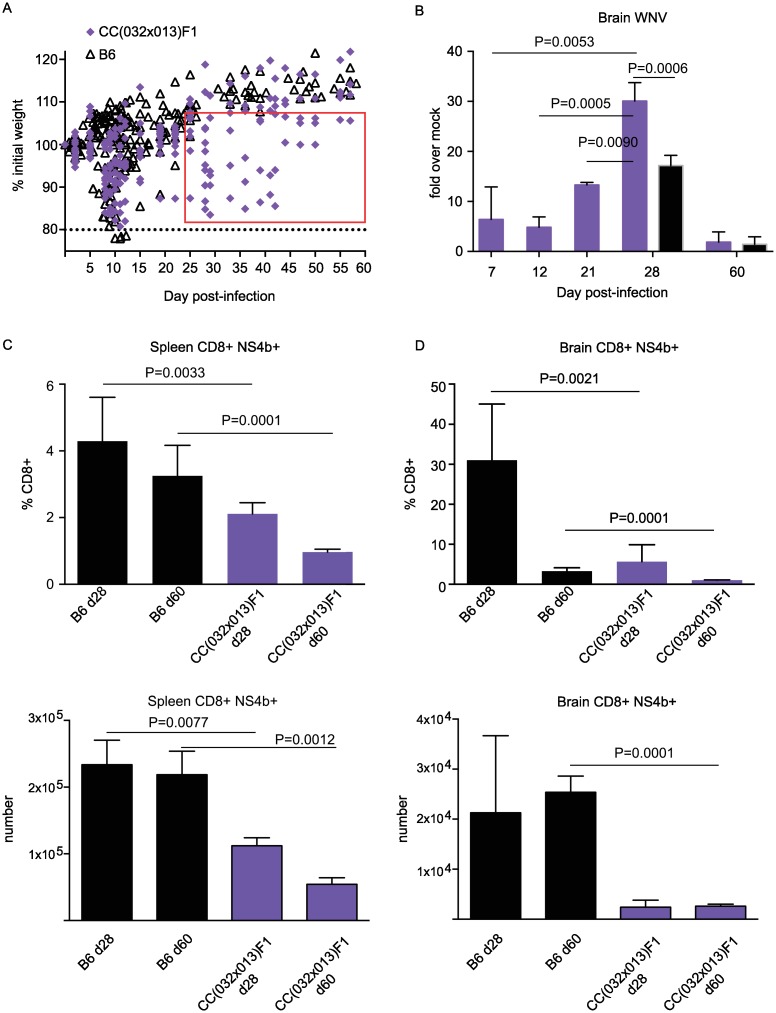
CC(032x013)F1 WNV-infected mice maintain a chronic WNV infection, with virus persisting in the CNS at d60. Cohorts of mice were infected with 100pfu WNV. (A) Compared to the B6 model of infection, CC(032x013)F1 mice do not return to and exceed starting weight by d25 p.i., but maintain weight loss out to d60. (B) PCR for WNV in brain at indicated time points. Results are from two separate experiments: the discovery screen, which included d7, d12, d21, and d28 time points, and validation mice, which included d28 and d60 endpoints. Results were comparable for overlapping endpoints. Viral RNA was detected in the brains of 66% of d28 and 20% of d60 mice, for both CC(032x013)F1 and B6 mice. (C) Spleen WNV-specific and (D) Brain WNV-specific CD8+ T cells in B6 and CC(032x013)F1 mice at corresponding time points. The discovery screen included three mice per time point (d7, 12, 21, and 28) and the validation experiments included six mice per time point.

As part of a screen of more than 100 RIX lines from the CC for unique disease outcomes resulting from West Nile virus infection, we identified CC(032x013)F1 as having a unique course of disease following WNV infection. B6 mice displayed the typical weight loss between days 7–14 p.i., with approximately 30% of animals succumbing to infection and the remainder recovering any weight lost by day 25 p.i. CC(032x013)F1 had a similar mortality rate of 38%, with 6/18 animals dying between days 10 and 13 p.i. and one dying on day 44 p.i. Notably, however, half of the mice surviving past d13 p.i. displayed sustained weight loss that was distinct from B6 mice (Fig 1A), as well as from the 100+ RIX lines screened [17]. Due to this novel and unique sustained weight loss following infection, we examined the viral burden within the CNS to determine if virus also persisted. While there were already high levels of WNV within the CNS at days 7 and 12 p.i., these levels increased dramatically out to day 28 p.i., and indeed virus persisted even at day 60 p.i. (Fig 1B). In accordance with this persistent virus present at later time points, there was a diminished WNV-specific CD8 T cell response in both the spleen and brain of CC(032x013)F1 compared to B6 mice, even at day 60 p.i. (Fig 1C and 1D).

**Fig 2:**
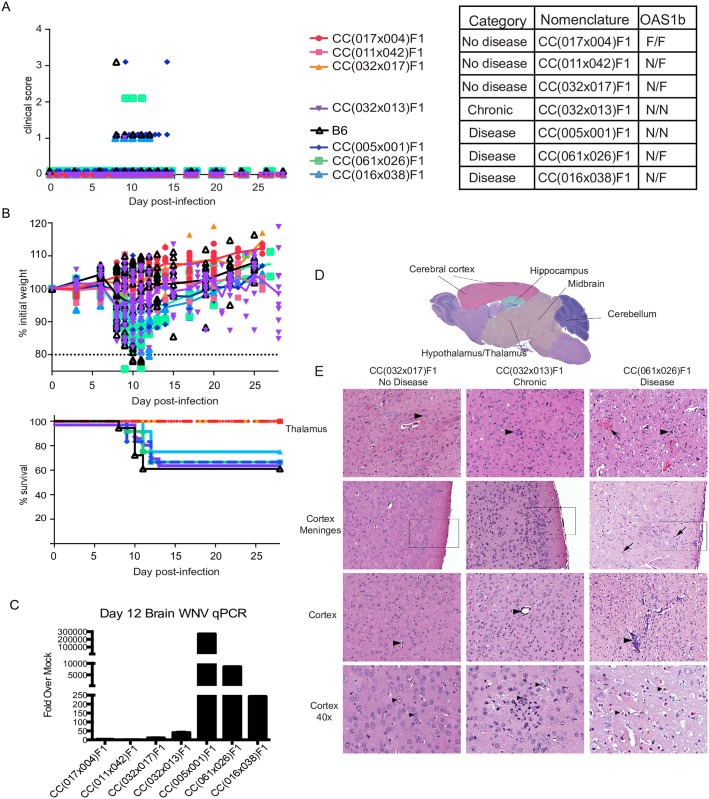
Comparison of WNV disease models by clinical presentation and neuropathology. (A) Representative RIX lines for 3 WNV disease model categories: 1) No disease 2) Chronic disease and 3) Disease. Representative “No Disease” and “Disease” lines were selected from 40+ lines of each category, identified through the screen. Each RIX line’s CC nomenclature and *Oas1b* allele status is shown (N = null, F = functional allele). Clinical scores and (B) weight loss and survival curves are shown for the seven CC RIX lines over the time course of WNV infection. “No disease” RIX show little to no weight loss and maintain a “0” clinical score across the time course. “Disease” RIX lines show increased weight loss by d8, as well as increase in clinical scores corresponding to neurological weakness and paralysis (see description in methods). (C) Brain WNV loads at d12 for the seven lines. p<0.001 for CC(032x013)F1 compared to all disease lines. (D-E) Neuropathology of WNV infection in RIX lines as indicated. (D) The approximate subregions of the brain that were scored for neuropathology are colorized. These regions were chosen due to the quality and consistency of the sections across the high throughput histologic study. (E) Hematoxylin and eosin stained sections of formalin-fixed paraffin-embedded tissues of WNV-infected lines as indicated with subregion noted. ***Thalamus***: Arrows indicate neurons. In the Chronic and Diseased tissues, the neurons are surrounded by glia (satellitosis). In the Disease tissue there is moderate hemorrhage (arrow), diffuse gliosis and mild malacia. ***Cortex Meninges***: In the Chronic and Diseased boxed region there is gliosis in the superficial cortex with mononuclear cell infiltrate in the meninges, which is more pronounced and widespread in the Diseased tissue. There are prominent mononuclear cells with rod-shaped nuclei in the Diseased cortex (arrows) are consistent with activated microglia. ***Cortex***: Arrowheads indicate blood vessels. In the Chronic and Diseased tissues there is mild to marked accumulation of mononuclear inflammatory cells in the vessel wall and perivascular space. In the Disease tissue there is moderate diffuse gliosis and perivascular cuffing. ***Cortex 40X***: Arrowheads indicate neuron cell bodies. In the Chronic tissue, there are glial nodules and early degeneration of neurons with mild malacia. In the Disease tissue the neurons are shrunken with dark eosinophilic cytoplasm, and pyknotic nuclei consistent with acute neuronal degeneration to apoptosis (left arrow). Note karyorrhectic debris (small basophilic bodies). All panels 20X except where indicated. Data represent three mice per group for all lines in the screen, and six mice per group for d28 and d60 validation time points.

Next, we compared CC(032x013)F1 to three CC-RIX lines that had no signs of disease (CC(017x004)F1, CC(011x042)F1, and CC(032x017)F1) and three RIX lines that did display traditional symptoms of disease as seen in the B6 model (CC(005x001)F1, CC(061x026)F1, and CC(016x038)F1) (Fig 2A). Determination of disease associated with infection was established by examination of both clinical score and weight loss over a 28-day time course (Fig 2A and 2B), as well as WNV in the brain quantified by PCR (Fig 2C), and histological examination of the brain (Fig 2D and 2E and S1 Fig). Specifically, RIX lines classified as having no disease experienced no clinical score, no weight loss or death, low to undetectable levels of WNV within the brain, and little to no histological scoring of brain tissues. In contrast, RIX lines with disease exhibited higher clinical scores, weight loss and/or death, detectable WNV present within the brain, and various signs of tissue destruction and/or inflammation within most or all regions of the brain detectable by histopathological examination of H&E stained sections of brain tissues (Fig 2 and S1 Table). These representative RIX lines with disease present similarly to the B6 model of infection, as can be seen with respect to clinical score, weight loss, and percent survival over the time course of infection (Fig 2A and 2B). Comparatively, CC(032x013)F1 also appeared to have disease, as we observed signs of disease by clinical scoring starting at day 8 p.i., weight loss, and positive disease scores within all sections of the brain by day 12 p.i. apparent by histopathology (Fig 2 and S1 Fig). Of note, differences in susceptibility to and disease outcome following WNV between CC-RIX lines was not due solely to *Oas1b* haplotype, which has previously been shown to be a susceptibility allele for WNV and other flaviviruses in mice [19,20]; one of the RIX lines with no disease has two copies of the wild-derived “functional” *Oas1b* allele (F/F), whereas two of them are heterozygous (N/F), one of the RIX lines with disease has two copies of the non-functional (truncated) allele present in classic inbred strains (N/N), whereas two of the RIX lines are heterozygous, and the chronic RIX is also homozygous for the non-functional classic inbred allele (Fig 2A). These data, and the fact that the B6 model, which also has 2 copies of the classic inbred allele, does not show chronic disease outcomes, lead us to conclude that differences in disease outcome across CC-RIX lines and the mechanism leading to chronic infection are not due solely to *Oas1b* status.

### Altered innate immune responses in lymphoid tissues and the CNS distinguishes CC(032x013)F1 from RIX lines with no disease and those with disease

**Fig 3:**
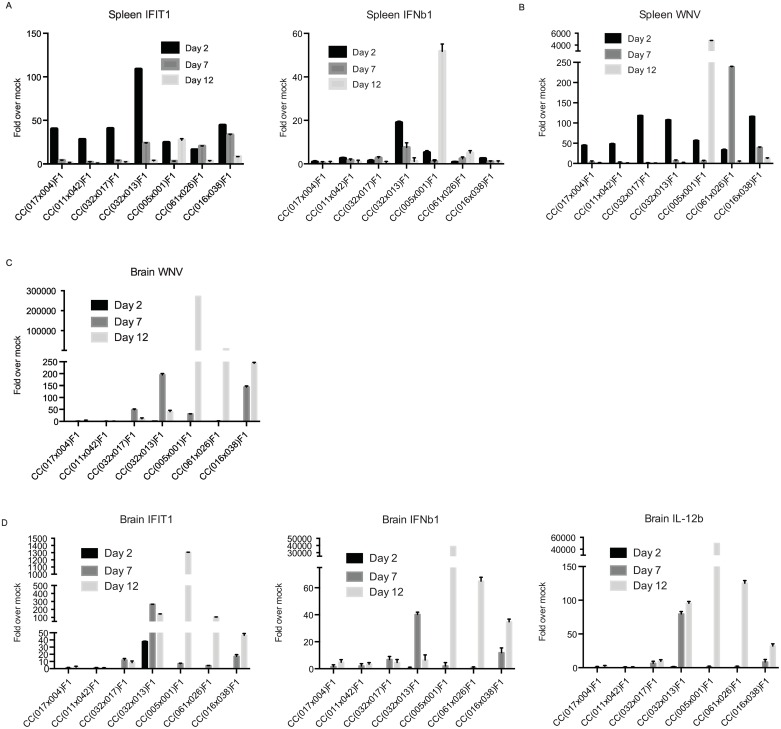
Differential innate immune signatures and viral load in the tissues. Total RNA was isolated from spleens and brains from CC RIX mice infected with WNV for the indicated times. Plots show qPCR results for *IFIT1* and *IFN-ß* expression (A) and WNV viral load in the spleen (B) and brain (C) as well as *IFIT1*, *IFN-ß*, and *IL-12* expression in the brain (D) at innate time points, relative to mock values. Data represent 3 mice per group at d2, 7, and 12 time points in the discovery screen. Fig 3A & B, p<0.001 for all comparisons of CC(032x013)F1 to the other RIX lines at d2 (*IFIT1* and *IFN-ß*); Fig 3C, p<0.01 for CC(032x013)F1 brain WNV load compared to the three non-disease lines at d7; Fig 3D, p<0.001 for all comparisons of CC(032x013)F1 to the other RIX lines at d2 (*IFIT1*); p<0.001 for all comparisons of CC(032x013)F1 to the other RIX lines at d7(*IFN-ß* and *IL-12*).

Because CC(032x013)F1 mice clearly exhibited a unique disease course following WNV infection as compared to the conventional inbred line B6 as well as the three RIX lines that showed no signs of disease and three RIX lines that did, we further examined the immune responses mounted upon infection in two target organs of WNV infection: the spleen, which notably is an immune inductive site as well as a target for virus replication, and the brain. First, innate immune activation was measured by assessing relative expression of interferon-induced protein with tetratricopeptide repeats 1 (*IFIT1*) and *IFN ß*. Both *IFNß* and *IFIT1* are IRF3-target genes induced by WNV infection, and *IFIT1* is further induced by IFN signaling, thereby allowing us to note induction of IRF-3 activation and IFN response. In the spleen, CC(032x013)F1 had a dramatically elevated *IFIT1* response two days p.i. compared to RIX lines with or without disease (Fig 3A). Interestingly, all three RIX lines with no disease had similar levels of *IFIT1* expression at day two p.i., with levels back to baseline by day seven, while RIX lines with disease showed persistence of this innate response, sometimes through day twelve p.i. (Fig 3A). Similarly, the *IFNß* response in the spleen was uniquely elevated at days two and seven p.i. in the chronic RIX, while there was not an appreciable *IFNß* response in the spleen of RIX lines with no disease at any of the time points examined (Fig 3A). Finally, the kinetics of virus expansion in the spleen was examined for all RIX. The RIX line with chronic disease appeared more similar to the no disease RIX lines in terms of WNV burden in the spleen, as there was an initial burst of virus replication in the spleen by day two p.i., followed by viral clearance, and little virus was detectable by day seven p.i. (Fig 3B). Conversely, in diseased RIX lines CC(005x001)F1 and CC(061x026)F1, there were extremely high loads of virus detected at days twelve and seven, respectively, and in CC(016x038)F1, virus remained detectable at day seven p.i. (Fig 3B), thereby demonstrating that WNV was not cleared quickly from the periphery in the diseased RIX lines as compared to no disease RIX lines and the chronic line, possibly due to differences in innate immune responses between RIX lines in these categories.

Next, we examined innate immune responses in the brain, as this is another target organ for WNV replication, and virus and immune involvement in this tissue likely results in disease symptoms. Here, little to no WNV was detectable at any time p.i. in RIX lines with no signs of disease. Surprisingly, however, there is high level of WNV present in the brain of mice from CC(032x013)F1 at day seven p.i., which is already much reduced by day twelve p.i. (Fig 3C). In contrast, the RIX lines with disease all have the highest WNV levels measured at day twelve p.i., suggesting that there is some level of control in the chronic RIX, although it is insufficient to lead to complete clearance even out to day 60 p.i. (Figs 1B and 3C). Uniquely, *IFIT1* expression in the brain of CC(032x013)F1 mice is rapidly elevated, with high responses detectable by day two p.i. and sustained at high levels through day twelve p.i., whereas in the other RIX lines with disease, expression does not rise above baseline until day seven or even twelve p.i. (Fig 3D). *IFNß* expression in the brain is also rapidly elevated in the chronic RIX by day seven p.i., though levels are near baseline by day twelve p.i., a time at which the diseased RIX lines are experiencing peak levels (Fig 3D). Finally, IL-12b levels, though not significantly elevated in the spleen p.i., are elevated early in the brain of CC(032x013)F1 mice, remaining high through day twelve p.i. (Fig 3D). Overall, it appears that the chronic RIX mounts a unique early innate immune response in both the spleen and brain following WNV infection, although this is not sufficient to prevent neuroinvasion or to clear virus from the brain to prevent chronicity of infection.

### A strong immunoregulatory signature distinguishes CC(032x013)F1 from CC-RIX lines with no disease

**Fig 4:**
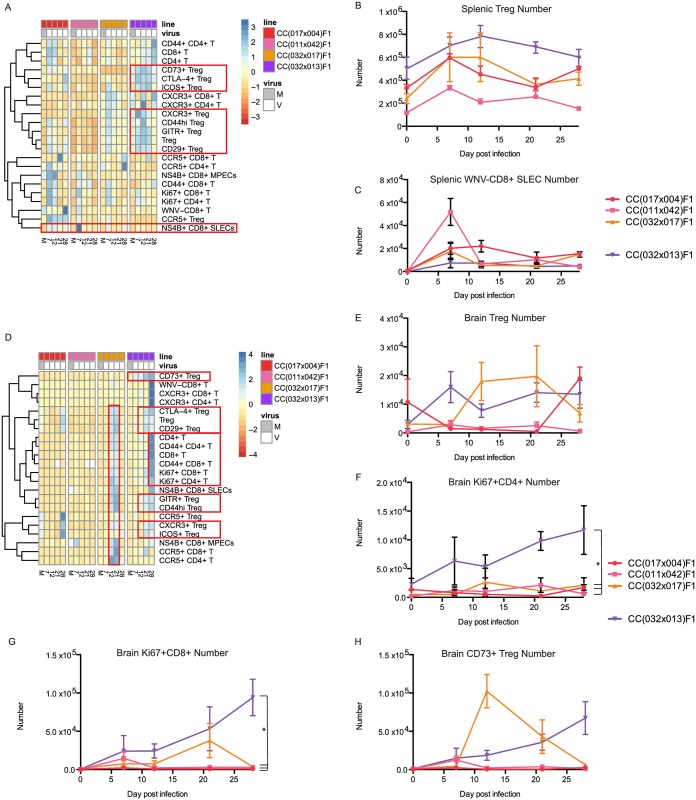
Flow cytometry heatmaps allow visualization of intra- and inter-strain comparisons of the immune response. (A) Three representative “no disease” lines are compared to the chronic line for immunophenotypes measured by mutli-parameter flow cytometry. Infection status and clinical observations are shown in the top module, while flow cytometry data is shown in the heat map below. T cell subsets and activation markers are shown (blue = up, red = down). Compared to the “no disease” RIX lines, the chronic RIX shows increased Treg numbers in the spleen, and increase in activation of those Tregs. Conversely, the chronic RIX shows a lack of expansion in WNV-specific short lived effector cells when compared to the no disease lines (red boxes). (B-C) Total number of splenic Tregs and WNV-CD8+ SLECs, as indicated. At baseline, p = 0.0176 for comparison of splenic Treg numbers from CC(032x013)F1 and CC(011x042), with all others non-significant. At d21 post-infection, differences in splenic Treg number were statistically significant for CC(032x013)F1 compared to CC(017x004)F1, CC(011x042)F1, and CC(032x017)F1 (p = 0.0440, 0.0159, and 0.0316, respectively). At d7 post-infection, p = 0.0232 for comparison of splenic WNV-CD8+ SLECs from CC(032x013)F1 and CC(011x042)F1, with all other comparisons p>0.05. (D) Flow cytometry heatmap of brain immunophenotypes from the same “no disease” and chronic lines. (E-G) Total number of brain Tregs, Ki67+ CD4 T cells, Ki67+ CD8 T cells, and CD73+ Tregs, as indicated. At d7 post-infection, differences in brain Treg number were statistically significant for CC(032x013)F1 compared to CC(017x004)F1, CC(011x042)F1, and CC(032x017)F1 (p<0.05). At d28 post-infection, differences in brain Ki67+ CD4+ T cell number were statistically significant for CC(032x013)F1 compared to CC(017x004)F1, CC(011x042)F1, and CC(032x017)F1 (p<0.05). N = 3 mice per group for all time points shown.

Due to the unique differences in innate immune responses observed in the spleen and brain of the chronic RIX line, we next examined the later stages of acquired immunity to WNV infection, with a focus on T cell responses using systematic multi-parameter flow cytometry. In particular, as we observed a unique and rapid innate response in the chronic RIX that is likely driving an inflammatory response yet a failure to prevent viral spread to or rapid clearance from the brain, we hypothesized that the resultant adaptive immune response may be dysregulated in CC(032x013)F1. In order to assess T cell signatures associated with protection from disease versus establishment of chronic infection, we used a systems biology heat map approach to both visualize our high-dimensional flow cytometry data, as well as identify those cell subsets differentially present in our chronic model. Strikingly, as compared to RIX lines with no disease, the chronic RIX CC(032x013)F1 mice displayed a strong and distinct immunoregulatory signature in the spleen starting early at day seven p.i., dominated by increased numbers of regulatory T cells (Tregs), as well as increased activation of these suppressive cells measured by expression of CD73, CTLA-4, ICOS, GITR, and CD44 as well as increases in Treg expression of CNS migration markers such as CXCR3 and CD29 (Fig 4A). In fact, the number of Tregs present in the spleen of CC(032x013)F1 was even elevated in naïve mice compared to RIX lines with no disease (Fig 4A and 4B), suggesting that increased splenic Treg numbers could pre-dispose the host to disease and perhaps even chronic WNV infection. In addition, the proportion of short-lived effector (SLEC) CD8 T cells, defined as WNV-specific by MHC class I tetramer staining as well as KLRG-1 expression, was increased at day 7 p.i. in all of the RIX lines with no disease but not in the RIX with chronic infection (Fig 4A and 4C). Thus, it appears that a rapid increase in the number of SLECs in the spleen upon WNV infection could serve as a correlate of protection from subsequent chronic disease.

In the brain, there was little T cell activity present p.i. in RIX lines with no disease, as predicted considering the general lack of virus present in this tissue. However, CC(032x017)F1, which does have low levels of virus present in the brain at day seven p.i. (Fig 3C), does have a T cell signature present at days 12 and 21 p.i. indicating the presence of a conventional T cell and Treg response, specifically consisting of increases in CD4 and CD8 T cells that express CD44+ and Ki67+, as well as increased Treg numbers and associated activation makers (Fig 4D–4G). As this RIX line was able to clear virus largely by day twelve p.i. (Fig 3C), it appears that the immune response in the brain was able to clear the infection without causing any overt signs of disease, and so this distinct brain signature can be considered to be uniquely protective and appropriately balanced. The RIX line with chronic disease, CC(032x013)F1, has a different immune signature including rapid increases in the number of CD73+ Tregs by day seven p.i. that persists out to day 28 p.i. (Fig 4D and 4H), as well as a slightly later immunomodulatory module including increased numbers of GITR+ Tregs, CD44hi Tregs, CD29+ Tregs, CTLA-4+ Tregs, CXCR3+ Tregs, and ICOS+ Tregs (Fig 4D). Finally, there is also a late signature, starting at day 21 p.i. and most strong at day 28, that includes an activated and proliferating CD4 and CD8 T cell response (Fig 4D–4F), likely in response to the later burst of virus replication observed at these time points (Fig 1B).

### An early increase in regulatory T cell numbers in the brain allows for establishment of WNV chronic infection over traditional disease course

**Fig 5:**
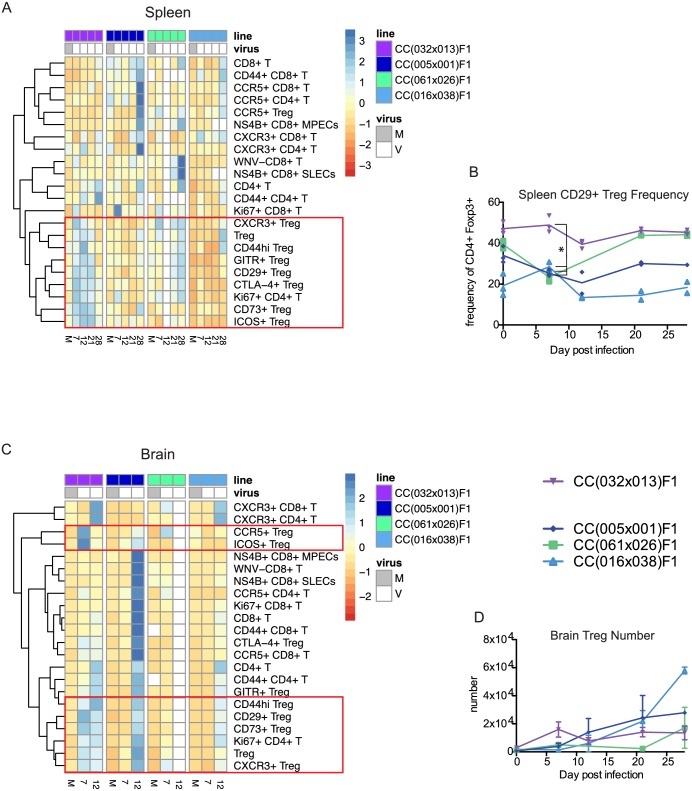
Chronic infection model mice show increased splenic Tregs over the time course of WNV infection compared to mice with disease. (A) Three representative RIX lines with disease are compared to the chronic line, with T cell subsets and activation markers measured by flow cytometry as indicated (blue = up, red = down); data from spleen. (B) CD29+ Treg frequencies in the spleen. At d7 post-infection, differences in spleen CD29+ Treg frequency were statistically significant for CC(032x013)F1 compared to CC(005x001)F1, CC(061x026)F1, and CC(016x038)F1 (p<0.005). (C) Flow cytometry analysis heat maps from brain samples for the same lines and time points. (D) Brain Treg numbers. N = 3 mice per group for all time points shown.

When comparing the chronic RIX to RIX lines with signs of disease, a distinct immunoregulatory signature in the spleen again marks the chronic RIX as unique compared to three RIX lines with disease (Fig 5A). This signature consists of increased numbers of splenic Tregs as well as Treg activation in terms of expression of CTLA-4, Ki67, CD44, GITR, CXCR3, CD29, CD73, and ICOS. More specifically, CD29, or β1 integrin, which heterodimerizes with α4 to form VLA-4 and has been demonstrated to be critical for translocation of T cells across the blood brain barrier, [21], is increased in expression on Tregs early post infection in the chronic RIX, but not in RIX lines with a traditional disease course (Fig 5B), suggesting that the suppressive capacity of Tregs cells are critical in setting mice up for chronic infection. The brains from mice of the chronic RIX are distinguished from those of the disease RIX lines by increased early numbers of Tregs and activated Tregs at day seven p.i. (Fig 5C and 5D), again suggesting that early immunoregulation provided by Tregs within the CNS allows for establishment of chronic WNV infection.

**Fig 6:**
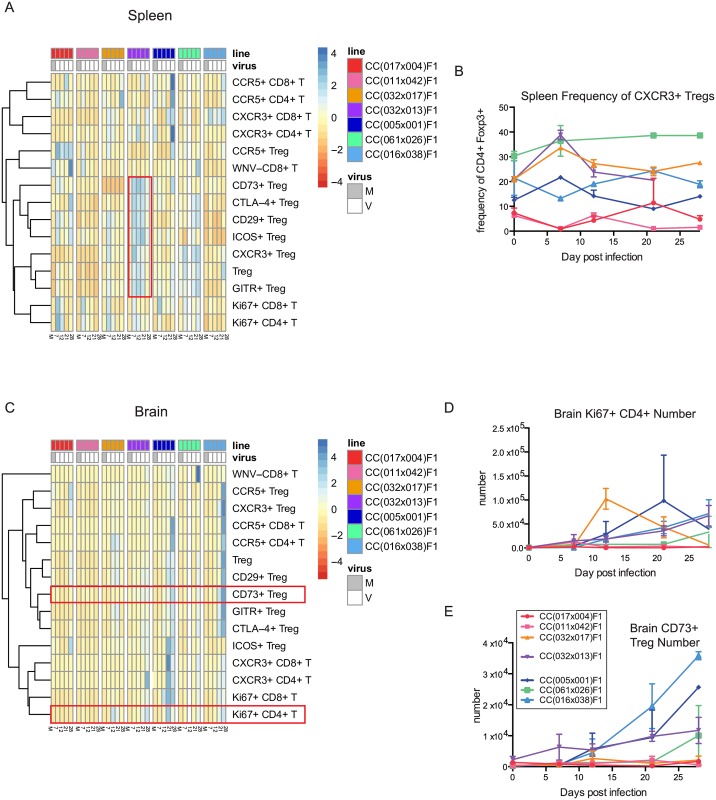
Flow cytometry visualization of the kinetics of the T cell response to WNV infection. (A) T cell subsets and activation markers are shown by heatmap for no disease, chronic, and disease model RIX lines over the time course of infection (spleen), and (B) frequency of CXCR3+ Tregs in the spleen. At baseline, differences in splenic frequency of CXCR3+ Tregs were statistically significant for CC(032x013)F1 compared to CC(017x004)F1 and CC(011x042)F1, (p<0.001). (C) T cell subsets and activation markers shown by heatmap in the brain, and (D) brain Ki67+ CD4 T cell numbers, and (E) brain CD73+ Ki67+ Treg numbers. N = 3 mice per group for all time points shown.

When the chronic RIX is compared head-to-head with RIX lines with no disease as well as those with traditional disease, there remains a consistent unique signature in the spleen of the chronic RIX, with a distinctive Treg activation module present in the spleen (Fig 6A, boxed in red). Furthermore, CXCR3 expression on Tregs, which is a chemokine receptor that allows for lymphocyte entry into the tissues, is elevated at baseline in all RIX lines that consequently have WNV entering the CNS, but not in the two lines that remained WNV-free in the brain (Fig 6B), suggesting that elevated CXCR3 expression on Tregs, and thus increased migratory Tregs, may be a correlate of susceptibility to CNS infection of WNV. Finally, when brain T cell responses are compared between chronically-infected mice and those with either no disease or traditional disease, we observed an early increase at day 12 p.i. in Ki67+ CD4 T cells in only in CC(032x017)F1 mice, the RIX line with no disease despite presence of WNV in the brain early p.i. (Figs 3C, 6C and 6D), suggesting that an early WNV-driven CD4 T cell response in the brain may protect mice from both traditional and chronic disease. Finally, a unique and early increase in CD73+ Tregs within the brain was present in the chronic RIX (Fig 6C and 6E), perhaps allowing for increased restraint of WNV-specific T cell responses and a resultant chronic infection. In sum, it appears that an immunoregulatory signature dominated by Tregs and Treg activation and migratory ability early p.i., or even prior to infection, in both the spleen and brain is key for establishment of chronic WNV infection.

### Mice chronically infected with WNV display a unique gene expression profile including reduced cytolytic ability

**Fig 7:**
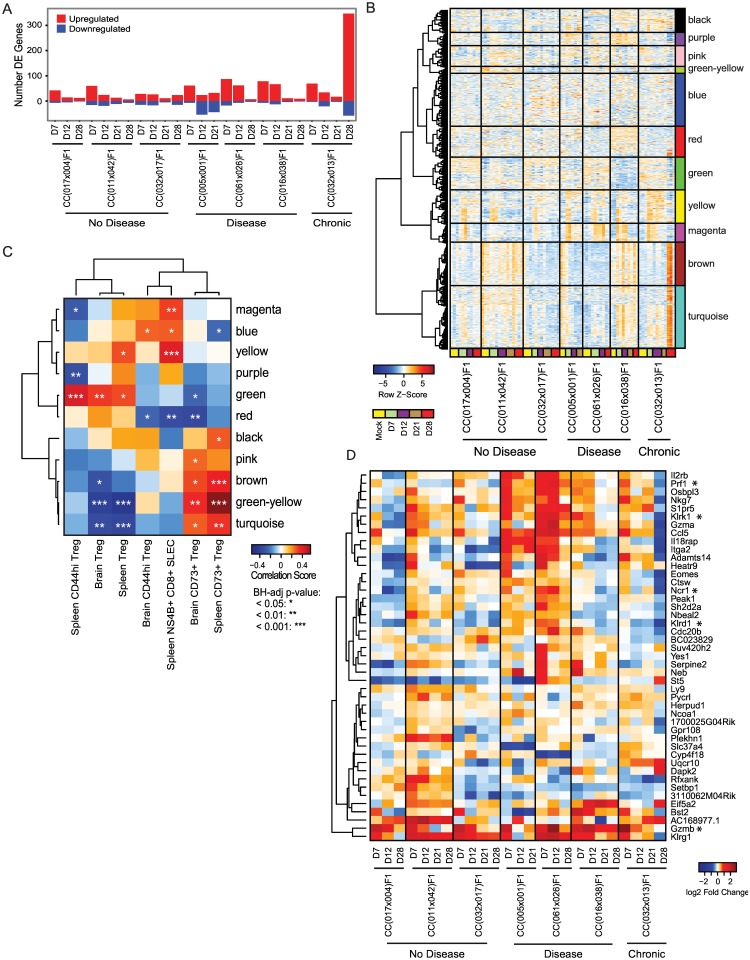
Mouse model of chronic WNV infection reveals that maintenance of cytolytic ability, associated with reduced activation of regulatory T cells, is critical for preventing viral persistence. (A) Microarry expression analysis of number of differentially expressed (DE) genes upregulated (red) or downregulated (blue) in each RIX across time as compared to mock-infected samples (Fold change > 1.5, BH-adjusted p-value < 0.05). (B) Normalized expression profiles of all genes found to be DE in at least one sample, visualized as relative level across all samples. Each column depicts an individual mouse, with the timepoint designated by a colored bar underneath. Genes were clustered by bicor correlation and divided into modules each labeled by an identifying color and their most highly significant Gene Ontology (GO) Biological Process (BH-adjusted p-value < 0.05). Module processes are color coded: black: GO:0030593 neutrophil chemotaxis, purple: GO:0019835 cytolysis, pink: GO:1990126 retrograde transport, endosome to plasma membrane, green-yellow: GO:0045444 fat cell differentiation, blue: GO:0043401 steroid hormone mediated signaling pathway, red: categories below significance threshold, green: categories below significance threshold, yellow: GO:0034976 response to endoplasmic reticulum stress, magenta: GO:0051607 defense response to virus, brown: GO:0006260 DNA replication, turquoise: GO:0006783 heme biosynthetic process. (C) Correlation map of module eigengenes to key flow cytometric immunophenotypes. Box color represents positive (red) or negative (blue) correlation score. Asterisks denote statistical significance of correlation. (D) Differential expression of genes within the “purple” (cytolosis) module as visualized by log_2_ fold change over mock-infected samples. Asterisks indicate genes directly involved in cytolytic and NK-killer cell cytotoxicity. Data shown are from three mice per group, for each time point.

Finally, we examined gene expression changes within the spleen following WNV infection in the chronic RIX versus three RIX lines with no signs of disease and three with signs of disease, via microarray analysis. Notably, there were considerably more differentially expressed (DE) genes in the chronic RIX at the day 28 p.i. time point, with the majority of the DE genes showing increases compared to naïve mice (Fig 7A). To expand this analysis, we examined expression profiles of all mice from these RIX lines across a variety of time points by using supervised WGCNA analysis to assess the underlying structure of the set of all differentially expressed genes. In this manner, we were able to examine the expression signatures of genes across each animal, taking into account the variation of genetic backgrounds and allowing us to focus on the pattern of gene regulation following WNV infection. Genes with common expression signatures, which are predicted to be under common regulatory nodes and to have similar functions, were clustered automatically and each labeled by the most highly enriched gene ontology (GO) biological process (Fig 7B). For many modules, relative expression of genes was temporally regulated following infection, such as the wave of upregulation and subsequent homeostatic control of the “magenta” module, with the top GO term of “defense response to virus” (Fig 7B). We next performed a correlation analysis of the eigengenes (1^st^ principle components) of the modules determined in Fig 7B and selected immunophenotypes identified by our flow cytometric analysis to be important for determining disease outcome following WNV infection. The pattern that each mouse displayed for each phenotype was compared across its transcriptomics profile, and a correlation score was calculated, as well as an associated p-value (Fig 7C). Interestingly, pathway analysis enrichment for the purple module revealed genes important for cytolysis by NK cells and CTLs (S2 Table), and overall across all RIX lines, genes in this module were less highly expressed when the frequency of CD44hi, antigen-experienced Tregs in the spleen was elevated (Fig 7C). This suggests a mechanism by which, upon exposure to WNV, Tregs restrain cytolytic cells. Furthermore, genes within this module were largely not increased following infection in the chronic RIX, whereas they were in the RIX lines that had traditional disease (Fig 7B), perhaps suggesting that diminished cytolysis is important for establishing chronic WNV infection in RIX lines where virus is not quickly controlled within the spleen without spread to the CNS (Fig 3B and 3C). Elevated gene expression in the magenta “defense response to virus” module is strongly correlated with the frequency of WNV-specific short-lived effector cells in the spleen, and negatively associated with the frequency of antigen-experienced Tregs in the spleen (Fig 7C), suggesting that genes within this module, many of which are important in pathogen-sensing and/or type I IFN signaling (categorized in S2 Table), assist in promoting an effective T-cell response in favor of Treg-mediated suppression. Finally, a heatmap depicting gene expression changes as log_2_ fold-changes over mock-infected samples from the purple “cytolysis” module reveals increased expression of cytolysis and NK cell cytotoxicity genes that is maintained over time following infection distinguishes RIX lines with traditional disease compared to chronic infection, as expression of *Prf1*, *Klrk1*, *Ncr1*, *Klrd1*, and *Gzmb* are decreased relative to uninfected animals at d28 p.i. in the chronic RIX, whereas expression is generally maintained at high levels compared to uninfected in the RIX lines with traditional disease (Fig 7D, S2 Fig and S2 Table). As this decreased cytolytic ability in the chronic RIX is preceeded by increased frequency of Tregs and Treg activation and migratory ability (Fig 5A), we hypothesize that this reduction in cytolytic ability is at least in part due to Treg-mediated suppression.

## Discussion

We report here for the first time a mouse model of chronic WNV infection that can be used to elucidate the immunological mechanisms underlying chronic flaviviral infection and disease in humans. By intercrossing inbred CC strains, we create outbred mice with replicable genomes. Among these lines, we identified CC(032x013)F1 hybrid males as having sustained weight loss and virus detectable in the brain out to day 60 p.i., as well as neuropathology within various regions of the brain. Interestingly, this RIX was able to largely clear virus from the spleen by d7 p.i. (Fig 3B), yet sustained brain viral loads for at least 2 months p.i. (Fig 1B). Clearance of the peripheral infection correlated with a rapid innate immune response within the spleen, including *IFIT1* and *IFNβ* expression that was elevated by day 2 p.i. at much higher levels than what was observed in the RIX lines without disease or in the RIX lines with the more traditional disease course (Fig 3A). In parallel with this robust innate immune response, however, the chronic RIX also has elevated Treg frequency and activation status, thereby suggesting that while the exuberant innate response allows for eventual peripheral clearance by day 7 p.i., it may also set up the host for a chronic infection at least in part due to enhanced immunoregulation. In the brain, the chronic CC(032x013)F1 also showed signs of a uniquely early innate immune response, though this was clearly not sufficient to quickly control virus replication in the CNS, perhaps because the innate immune expression of *IFNß* is negligible by d12, a time at which the response is just ramping up in the traditional disease RIX lines (Fig 3D). While *IL-12* expression in CC(032x013)F1 is quickly elevated within the brain, this inflammatory and T_H_1-promoting response is not able to completely suppress virus replication, perhaps due to immunoregulation mediated by Tregs. Taken together, we find that chronic disease following WNV infection is defined by several components, including sustained weight loss, altered innate immunity, and increased regulatory T cell frequency; we also find that chronically infected mice have lower expression of genes associated with cytolysis.

This battle mediated by inflammation and immunity induced by microbe replication versus immunoregulation to prevent collateral tissue damage has a well-documented history of resulting in chronic infections. For example, *Leishmania major* infection leads to an expansion of Tregs that allows for establishment of chronic infection, and the long-term exposure of antigen allows for the generation of strong protective memory responses upon subsequent re-exposure [22]. In these studies, mice which generated complete sterilizing immunity during primary infection due to deficiency in Treg IL-10 production were not protected against secondary challenge, implicating a role for host-pathogen co-evolution to promote mutual survival [22]. In studies of another chronic microbial infection, human peripheral and pleural Tregs were shown to similarly respond and expand upon exposure to *M*. *tuberculosis* [23–25], and several groups have reported a correlation between chronically elevated frequencies of Tregs in tuberculosis patients with active disease, compared to far lower frequencies in individuals who resolve infection, or who were never infected [23,26,27]. Hepatitis C Virus (HCV) can result in either viral clearance or chronicity, and it appears that the relative vigor of the acute immune response to infection controls disease outcome. A robust and early T_H_1 response is thought to be sufficient to clear acute infection, however decreases in T_H_1 intensity, or T_H_2 skewing tends to result in chronicity [28,29]. Only 30% of individuals are able to acutely control HCV infection [30], and while it has been hypothesized that CD8+ T cell exhaustion and the loss of CD4+ T cell help may together be responsible for the inability to clear virus [29], there is also evidence to suggest that a shift to favor Treg induction promotes viral persistence for both HBV and HCV [31–36]. In support of this hypothesis is the finding that the proportion of Tregs is increased in chronically infected individuals in both the liver and periphery [29,31–40]. It has been hypothesized that the host may induce the shift to Treg cell production in an attempt to mitigate cellular damage caused by the robust primary anti-viral immune response. Taken together, Tregs in the context of HCV infection arguably provide the host both benefit and detriment, by permitting long-term chronicity, but also protecting from excessive liver damage and hepatocellular carcinoma [32].

Previous studies that have interrogated the role for Tregs in clearing neurotropic viruses from the central nervous system and brain similarly highlight the necessity for a regulated balance of effector and Treg responses. For example, sterilizing immunity against WNV requires the recruitment of CD8+ T cells into the CNS [41], although these responses must be regulated to avoid excessive damage to non-renewable neurons. In agreement with this hypothesis, individuals who remain asymptomatic during WNV infection have an increased frequency of Tregs in peripheral blood, as compared to individuals with more severe disease, and Treg ablation in mice resulted in an increase in disease severity, weight loss, and lethality [42]. Here, we extend these previous findings to demonstrate for the first time that enhanced Treg frequency, activation, and tissue-migratory capacity in combination with other immune factors such as a robust and early innate immune response can lead to a chronic WNV infection. Further, we hypothesize that due to this enhanced immunoregulation provided by Tregs, there is reduced cytolysis by NK cells and/or CTLs at the later stages of infection, likely allowing for viral persistence within the brain. Previous studies have demonstrated the importance of both CTL and NK cell responses for WNV clearance from the CNS [41,43], suggesting that diminished expression of genes such as *Gzmb* and *Prf1* (Fig 7D) at day 28 p.i. in the chronic RIX may at least in part allow for viral persistence though reduced anti-viral effector cell function.

In sum, we describe here our novel discovery of a mouse model of chronic WNV disease generated from an F1 cross of two CC strains. While we have characterized the course of disease as well as the immune responses in this RIX that are associated with this chronic infection, future studies will reveal the underlying genetic polymorphisms contributing to chronic WNV infection and disease. The CC(032x013)F1 line is capable of supporting chronic WNV infection, will be useful to further elucidate the mechanisms that allow for chronicity, and further, could be used to evaluate disease in other viral infections that lack good mouse models such as Zika virus, and to investigate putative therapies to curtail chronic infections.

## Materials and Methods

### Virus

West Nile virus TX-2002-HC (WN-TX) was propagated as previously described (26). 100 PFU WN-TX was used as infectious dose in all studies.

### CC RIX lines and disease definitions

The clinical scoring system to evaluate WNV-infected mice is as follows: 0 = healthy mouse (baseline), 1 = ruffled fur, lethargy, hunched posture, no paresis, normal gait, 2 = altered gait, limited movement in 1 hind limb, 3 = lack of movement, paresis in 1 or both hind limbs, 4 = moribund. Based on weight loss, clinical scoring, and brain histology, CC RIX lines segregated into three broad categories: No disease, Chronic, and Disease. Mice with disease are defined as having a weight loss greater than or equal to 10% and/or any death, whereas no disease mice are defined by a weight loss of less than 10% and no death. The CC dRIX lines featured in this study are depicted in Fig 2A.

### Mice, infection, and experimental procedures

CC RI mice were obtained from the Systems Genetics Core Facility at UNC [44]. For the screen, CC RIX lines were bred at the University of North Carolina-Chapel Hill (UNC) under specific pathogen free conditions. 6–8 week old F1 hybrid male mice were transferred from UNC to the University of Washington and housed directly into a BSL-2+ laboratory within an SPF barrier facility. Age- and sex-matched eight to ten week old mice were subcutaneously inoculated in the rear footpad with 100 PFU WN-TX. Mice were monitored daily for morbidity (% of initial weight loss) and clinical disease scores. Mice were housed in BL3 conditions throughout the experiments and tissues were processed under BSL3 conditions. Genotypes of interest in this study, *H2b* and *Oas1b*, were obtained from the Systems Genetics Core Facility at UNC [44]. For the discovery screen, mice were infected and monitored as described above, and then sacrificed at days 2, 4, 7, and 12 (for innate immune studies) or days 7, 12, 21, and 28 (for adaptive immune studies). These data include qPCR for WNV RNA and protein expression, histology, flow cytometry analysis, microarray gene expression analysis, and transcriptomic analysis, as further detailed below.

### Histology

Mock-infected or WNV-infected mice were sacrificed, exsanguinated, and perfused with PBS-4% paraformaldehyde, pH 7.3. Brains were embedded in paraffin and 4–6 μm sections were prepared and stained with hematoxylin and eosin (H&E) by the UW Histology and Imaging Core. H&E-stained sections were evaluated for viral-induced neuropathology in five general regions of the brain (cortex, hippocampus, hypothalamus/thalamus, midbrain, and cerebellum). Lesions were scored for severity on a semi-quantitative scale for perivascular inflammation, parenchymal inflammation, hemorrhage and neuronal necrosis (0–4; see S1 Table).

### RNA extraction and analysis procedures

Spleen, kidney and brain were removed from mock infected or WNV infected mice after perfusion as described above. Organs were suspended in RNA-later and stored, following which they were suspended in PBS, homogenized using a Precellys 24 machine at 1500 RPM for 20 seconds (Bertin Technologies, France). Total RNA was extracted using the RiboPure Kit (Ambion), and cDNA was synthesized with the iScript Select DNA Synthesis Kit (Biorad) and evaluated for WNV using a probe specific for the E gene, IFIT1, IL-12b, and IFN-β1 RNA expression by SYBR Green RT-qPCR. The qPCR for WNV was based on the 2-ΔΔCT method and normalized for the individual GAPDH values in each sample. The values represent the fold over mock increase in signal for the WNV E gene over an arbitrarily low value in the mock that represents a virus-null sample. Specific primer sets are:

WNV: 5’TCAGCGATCTCTCCACCAAAG, 3’GGGTCAGCACGTTTGTCATTG

mIFIT1: 5’CTGAGATGTCACTTCACATGGAA, 3’GTGCATCCCCAATGGGTTCT

mIFNb1: 5’CAGCTCCAAGAAAGGACGAAC, 3’GGCAGTGTAACTCTTCTGCAT

mIL-12b: 5’TGGTTTGCCATCGTTTTGCTG, 3’ACAGGTGAGGTTCACTGTTTCT

### Affymetrix Target Preparation and Microarray Hybridization

RNA samples were prepared for whole transcriptome expression analysis using the WT PLUS Reagent Kit following the manufacturer’s recommended protocol (Affymetrix, Inc.). 100 ng total RNA was used to prepare the hybridization ready targets. Individual sense-strand DNA targets were randomized and hybridized to Mouse Gene 2.1 ST 24-Array Plates (Affymetrix, Inc.) using the GeneTitan Multi-Channel (MC) Instrument for hybridization, staining and washing of arrays, as well as for scanning. Quality control (QC) metrics for hybridization, labeling and sample quality were generated using the Affymetrix Expression Console (version 1.3.187) software. All samples passed QC criteria.

### Transcriptomic analysis

Samples were screened for QC and outlier detection using the Affymetrix expression console using boxplots, as well as multi-dimensional scaling (MDS) analysis and inter-array correlation (IAC) plots using the R statistical programming language (version 3.1), Bioconductor (version 2.13) and packages oligo (1.32.0), oligomask (https://github.com/dbottomly/oligoMask), limma (3.28.4), and corresponding dependencies. Background correction, normalization, probe masking and probe summarization were performed with RMA (Robust Multichip Average) within the oligomask package. The sva package (3.20) in R was used to correct for plate batch effects. Differential expression (DE) analysis was performed using the limma package, with a threshold of significance of a > 1.5 fold change over mock with a Benjamini-Hochberg-adjusted p-value < 0.05. The union of all DE genes for at least one timepoint in at least one RIX was used as a gene set for immunophenotypic correlation analysis. The normalized array signals of these genes for each sample were visualized by clustergram colored relative to row Z-scores. Clustering was performed using the bicor function within the WGCNA package (1.51), and the dendrogram was cut using the cutreeStatic option of the dynamicTreeCut package (1.63–1). Module eigengenes were calculated by multiplying the first elements of the SVD decomposition. Correlations between modules and phenotypes were calculated using the bicor method. Heatmaps were created using the heatmap.2 function of gplots (3.0.1). Numbers of DE genes were plotted using ggplot2 (2.1.0). Data were deposited in the GEO repository (access number GSE82046).

### CC-probe masking

To ensure that all Affymetrix probes work properly across all CC lines, previously described masking techniques were applied based off the CC founder data. To mask out problematic Affymetrix probes, we used the oligomask R package designed for CC mouse data [45]. Oligomask uses VCF files and the oligo package to filter probes prior to normalization and statistical analysis.

### Functional Network Analysis

Genes detected in the purple module were loaded into Ingenuity Pathway Analysis (IPA) software for enrichment of biological pathways. The top two canonical pathways (Score 27 and 24) were merged and displayed as a network. This network is associated with cell death, survival, cellular compromise, and tissue morphology. The network was then imported into IPA’s pathway designer and displayed using the subcellular setting to reveal the orientation of genes within the cell. Gene colors represent relative expression on day 28 in the chronic RIX.

### Cell preparation for flow cytometry assays

Following euthanasia, mice were perfused with 10 ml PBS to remove any residual intravascular leukocytes. Spleens were homogenized, treated with ACK lysis buffer to remove red blood cells, washed, and resuspended in FACS buffer (1X PBS, 0.5% FBS). To obtain lymphocytes from the CNS, brains were harvested into RPMI and a suspension created through mechanical disruption. The suspension was added to hypertonic Percoll to create a 30% Percoll solution, vortexed, and centrifuged at 1250 rpm for 30 minutes at 4°C. After aspirating the supernatant, any remaining red blood cells in the cell pellet were ACK lysed, washed, passed through a 70 μm nylon mesh, and resuspended in FACS buffer. Cells were counted by hemacytometer using trypan blue exclusion.

### Flow cytometry analysis

Following preparation of single cell suspensions, cells were plated at 1 x 10^6^ cells/well and stained for surface markers for 15 minutes on ice. For tetramer staining, cells were stained with the WNV NS4b-H2D^b^ tetramer (generated by the Immune Monitoring Lab, Fred Hutchinson Cancer Research Center). Cells were subsequently fixed, permeabilized (Foxp3 Fixation/Permeabilization Concentrate and Diluent, Ebioscience) and stained intracellularly with antibodies for 30 minutes on ice. Flow cytometry was performed on a BD LSRII machine using BD FACSDiva software. Analysis was performed using FlowJo software. The following directly conjugated antibodies were used: CD3-ECD (143-2C11), CD4-BV605 (RM4-5), CD8-BV650 (53–6.7), Foxp3-Alexa700 (FJK-16S), NS4b class I tetramer-APC, CD44-FITC (IM7), CD73-BV421 (TY/11.8), CTLA-4-APC (UC10-4B9), ICOS-PECy5 (7E.17G9), CXCR3-PerCP eFlour710 (CXCR3-173), GITR-PECy7 (DTA-1), CD29-APC Cy7 (HMb1-1), CCR5-PE (HM-CCR5), Ki67-FITC (SolA15), KLRG-1-PECy7 (2F1), and CD127-PECy5 (A7R34). AmCyan Live/dead stain (Invitrogen) was used in all panels for identification of live cells. Flow cytometry heatmaps were created with the pheatmap package (http://CRAN.R-project.org/package=pheatmap) in the R statistical computing environment (http://www.R-project.org/). The values plotted are z-scores of the counts for each cell population.

### Statistical analysis

When comparing groups, two-tailed unpaired Student’s *t* tests were conducted, with p-values <0.05 considered significant. Error bars show +/- SEM or +/- SD (Fig 1C and 1D).

### Ethics Statement

All animal experiments were approved by the University of Washington Institutional Animal Care and Use Committee. The Office of Laboratory Animal Welfare of the National Institutes of Health (NIH) has approved UNC (#A3410-01) and the University of Washington (#A3464-01), and this study was carried out in strict compliance with the Public Health Service (PHS) Policy on Humane Care and Use of Laboratory Animals. All experiments were carried out according to approved UW IACUC protocols (#4327–01 and 4158–01).

## Supporting Information

S1 Fig Neuropathology of representative CC-RIX lines. Neuropathology of WNV infection in CC lines as indicated, assessed by H&E scoring.
(TIF)

S2 Fig Network based off the genes found in the “purple” module, as depicted in Fig 7. (TIF)

S1 Table Histology Scoring Methods. (TIF)

S2 Table Pathway analysis enrichment for Purple, Magenta, Green-Yellow, and Yellow Modules. (PDF)
