# Supplementary figures and images for: A Mouse Model of Chronic West Nile Virus Disease
Source: PLoS Pathog. 2016 Nov 2;12(11):e1005996. doi: 10.1371/journal.ppat.1005996 (PMC5091767; doi:10.1371/journal.ppat.1005996)

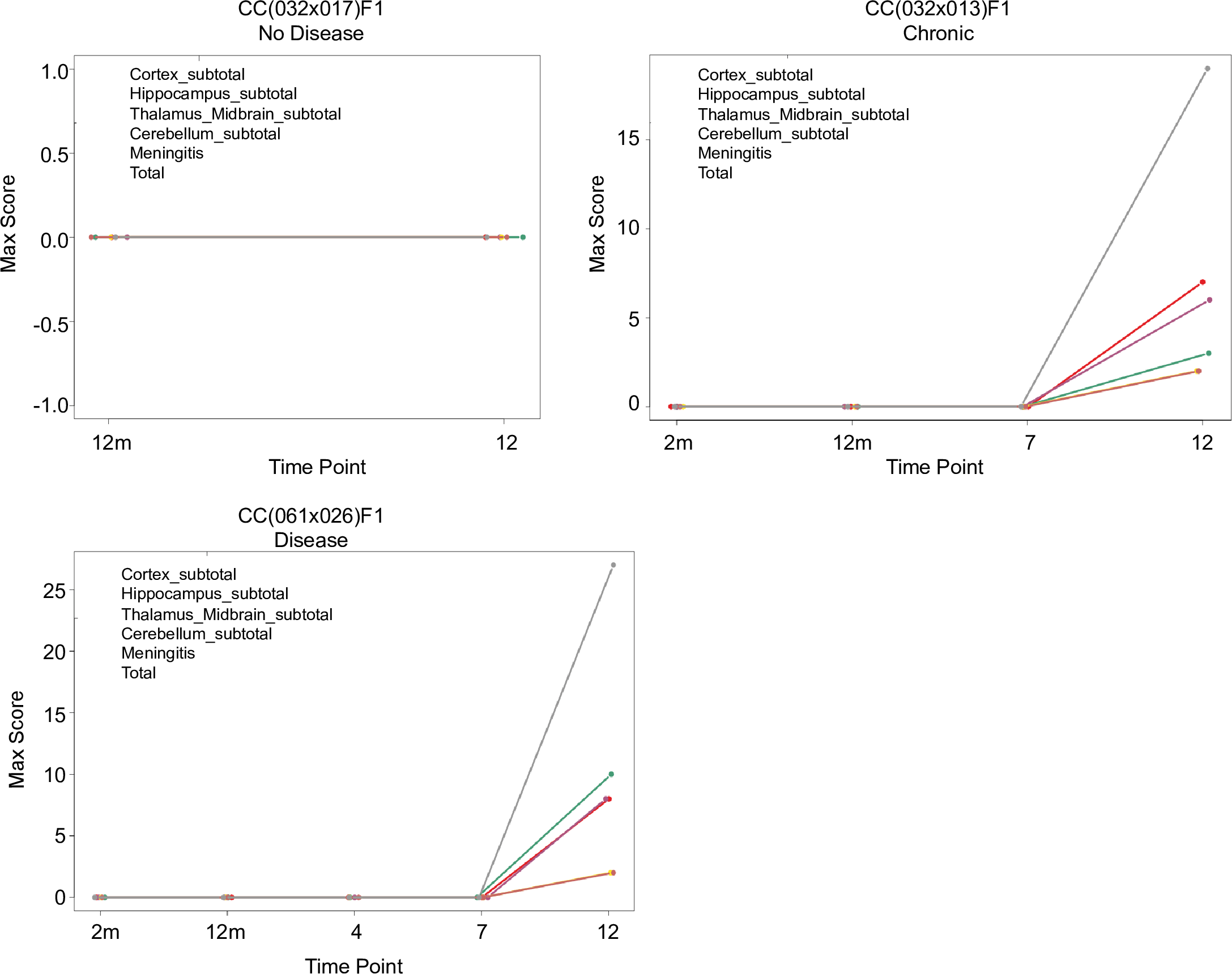

Supplement: S1 Fig — Neuropathology of WNV infection in CC lines as indicated, assessed by H&E scoring. (TIF) [file ppat.1005996.s001.tif]

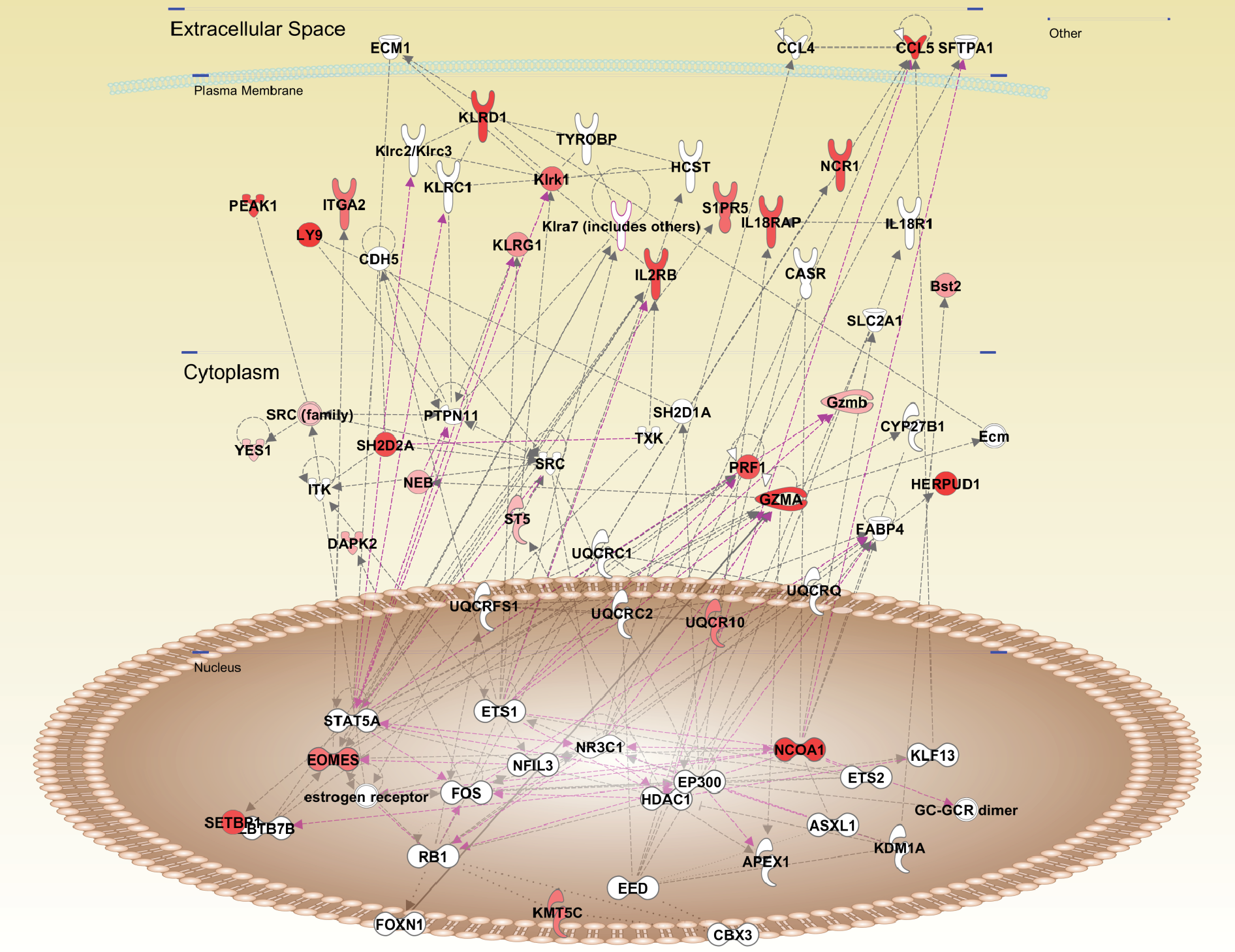

Supplement: S2 Fig — (TIF) [file ppat.1005996.s002.tif]

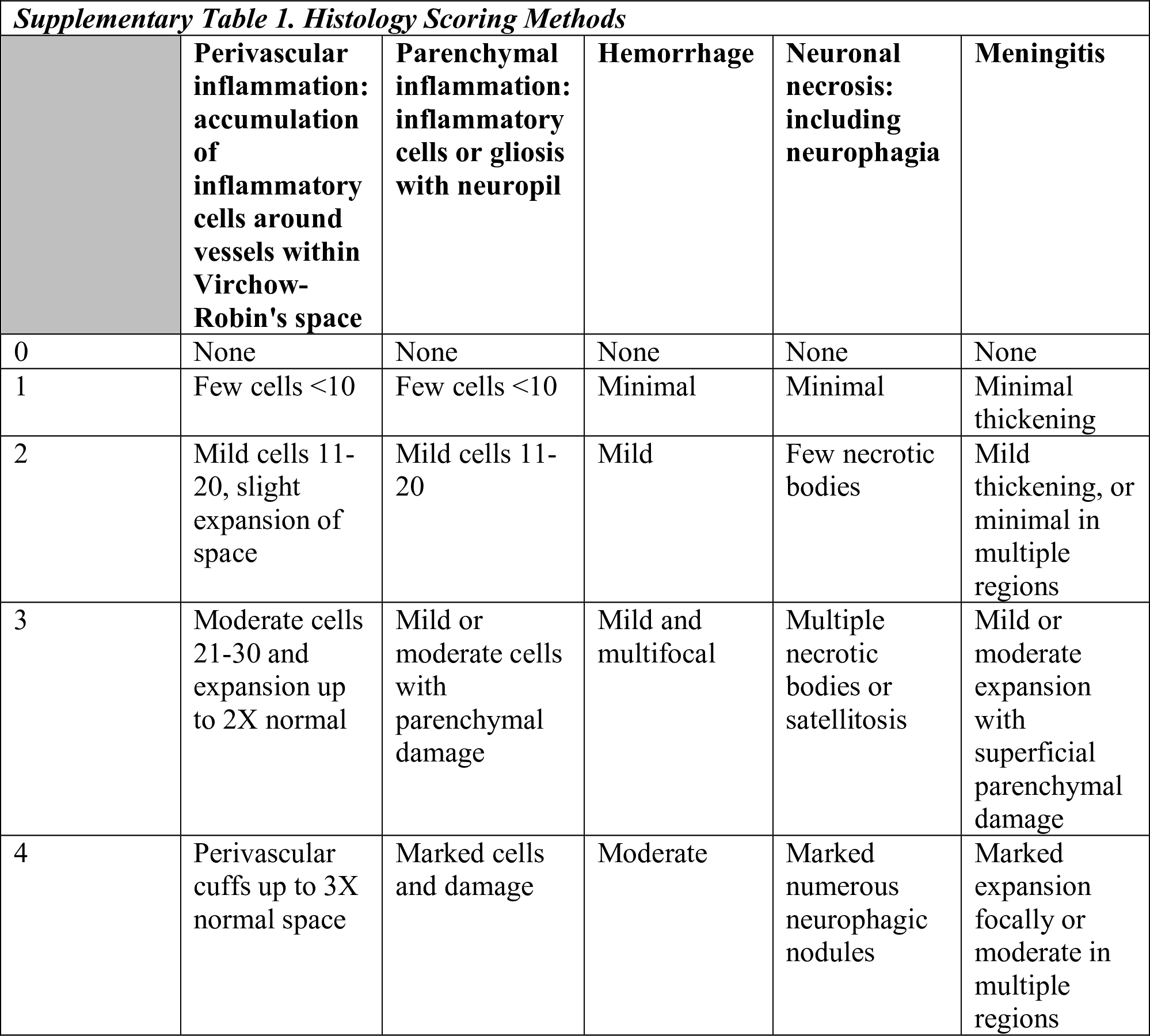

Supplement: S1 Table — (TIF) [file ppat.1005996.s003.tif]
