# Supplementary material for: A Mouse Model of Chronic West Nile Virus Disease
Source: PLoS Pathog. 2016 Nov 2;12(11):e1005996. doi: 10.1371/journal.ppat.1005996 (PMC5091767; doi:10.1371/journal.ppat.1005996)
Supplement: S2 Table — (PDF) [file ppat.1005996.s004.pdf]

Supplementary Table 2. Pathway analysis enrichment for Purple, Magenta, Green-Yellow, and Yellow Modules.					
**Module**	**Pathway name**	**Pathway uploaded gene count**	**Pathway p-value**	**Pathway p-value corrected**	**Gene symbols**
Purple	Natural killer cell mediated cytotoxicity	5	7.45E-06	2.31E-04	Gzmb; Klrd1; Klrk1; Ncr1; Prf1;
	Graft-versus-host disease	3	2.48E-04	0.003841339	Gzmb; Klrd1; Prf1;
	Cytochrome P450 - arranged by substrate type	2	0.010995312	0.048693526	Cyp4f18; Ncoa1;
	Allograft rejection	2	0.006302683	0.048845793	Gzmb; Prf1;
Magenta	Hepatitis C	10	1.42E-12	4.53E-11	Eif2ak2; Gm14446; Ifit1; Irf7; Irf9; Oas1a; Oas1g; Oas2; Oas3; Stat1;
	Interferon Signaling	8	1.33E-11	2.12E-10	Ifit1; Irf7; Irf9; Oas1a; Oas2; Oas3; Oasl1; Usp18;
	Interferon gamma signaling	6	1.58E-09	1.69E-08	Irf7; Irf9; Oas1a; Oas2; Oas3; Oasl1;
	Immune System	14	1.53E-08	1.23E-07	C2; Dtx3l; Ifih1; Ifit1; Irf7; Irf9; Oas1a; Oas2; Oas3; Oasl1; Tlr7; Trim25; Usp18; Zbp1;
	Cytokine Signaling in Immune system	8	3.85E-08	2.46E-07	Ifit1; Irf7; Irf9; Oas1a; Oas2; Oas3; Oasl1; Usp18;
	TRAF3-dependent IRF activation pathway	3	4.41E-06	2.35E-05	Ifih1; Irf7; Trim25;
	TRAF6 mediated IRF7 activation	3	3.03E-05	1.38E-04	Ifih1; Irf7; Trim25;
	RIG-I-like receptor signaling pathway	4	3.47E-05	1.39E-04	Dhx58; Ifih1; Irf7; Trim25;
	NF-kB activation through FADD/RIP-1 pathway mediated by caspase-8 and -10	2	3.45E-04	0.001227619	Ifih1; Trim25;

	RIG-I/MDA5 mediated induction of IFN-alpha/beta pathways	3	5.67E-04	0.001814866	Ifih1; Irf7; Trim25;
	Cytosolic DNA-sensing pathway	3	6.58E-04	0.001913587	Adar; Irf7; Zbp1;
	Interferon alpha/beta signaling	2	7.99E-04	0.001965845	Irf9; Usp18;
	TRAF6 mediated IRF7 activation in TLR7/8 or 9 signaling	2	7.99E-04	0.001965845	Irf7; Tlr7;
	Antiviral mechanism by IFN-stimulated genes	2	0.00143258	0.002865161	Ifit1; Usp18;
	ISG15 antiviral mechanism	2	0.00143258	0.002865161	Ifit1; Usp18;
	TRAF6 mediated NF-kB activation	2	0.00158062	0.002975284	Ifih1; Trim25;
	Innate Immune System	6	0.001346877	0.003078576	C2; Ifih1; Irf7; Tlr7; Trim25; Zbp1;
	Toll-like receptor signaling pathway	3	0.002746939	0.004626423	Irf7; Stat1; Tlr7;
	Negative regulators of RIG-I/MDA5 signaling	2	0.002614411	0.004647841	Ifih1; Trim25;
	Cytosolic sensors of pathogen-associated DNA	2	0.014502922	0.023204675	Irf7; Zbp1;
	MyD88 dependent cascade initiated on endosome	2	0.019982214	0.029065038	Irf7; Tlr7;
	Toll Like Receptor 7/8 (TLR7/8) Cascade	2	0.019982214	0.029065038	Irf7; Tlr7;
	Toll Like Receptor 9 (TLR9) Cascade	2	0.021468569	0.029869314	Irf7; Tlr7;
Green-yellow	Initial triggering of complement	2	1.57E-04	0.001415559	C1qb; Cfp;
	Complement cascade	2	4.99E-04	0.002246038	C1qb; Cfp;
	Amino sugar and nucleotide sugar metabolism	2	0.00133385	0.003001163	Gnpda1; Renbp;
	Innate Immune System	4	0.001115641	0.003346924	C1qb; Cfp; Myo10; Tlr4;
	Chagas disease (American trypanosomiasis)	2	0.005875128	0.010575231	C1qb; Tlr4;
	Immune System	4	0.013038267	0.019557401	C1qb; Cfp; Myo10; Tlr4;
	Metabolism of lipids and	3	0.018020105	0.023168707	Abcc3; Lpin1; Ptgs1; c

	lipoproteins				
Yellow	Protein processing in endoplasmic reticulum	14	3.95E-14	3.52E-12	Ckap4; Derl3; Edem1; Edem2; Hsp90b1; Hspa5; Hyou1; Lman1; Pdia6; Sec24d; Sel1l; Ssr2; Txndc5; Xbp1;
	ATF6-alpha activates chaperone genes	3	1.47E-05	4.37E-04	Hsp90b1; Hspa5; Xbp1;
	Unfolded Protein Response (UPR)	5	1.15E-05	5.10E-04	Edem1; Hsp90b1; Hspa5; Hyou1; Xbp1;
	ATF6-alpha activates chaperones	3	2.68E-05	5.96E-04	Hsp90b1; Hspa5; Xbp1;
	Malaria	4	9.14E-05	0.001626369	Itgb2; Met; Myd88; Tnf;
	Tristetraprolin (TTP) destabilizes mRNA	2	1.53E-04	0.002270271	Mapkapk2; Zfp36;
	Metabolism of proteins	10	3.99E-04	0.005071064	Ace; B4galt5; Edem1; Fut8; Hsp90b1; Hspa5; Hyou1; Lman1; Ssr2; Xbp1;
	Transport to the Golgi and subsequent modification	3	5.11E-04	0.005687354	B4galt5; Fut8; Lman1;
	IRE1alpha activates chaperones	3	0.001255271	0.011171914	Edem1; Hyou1; Xbp1;
	XBP1(S) activates chaperone genes	3	0.001169782	0.011567842	Edem1; Hyou1; Xbp1;
	Asparagine N-linked glycosylation	4	0.001710341	0.013838216	B4galt5; Edem1; Fut8; Lman1;
	Leishmaniasis	3	0.004211903	0.026775672	Itgb2; Myd88; Tnf;
	N-glycan antennae elongation in the medial/trans-Golgi	2	0.004153767	0.028437325	B4galt5; Fut8;
	Toll-Like Receptors Cascades	4	0.003930678	0.02915253	Hsp90b1; Lbp; Mapkapk2; Myd88;
	Asthma	2	0.006066244	0.035993046	Prg2; Tnf;
	Antigen processing and presentation	3	0.006564321	0.036514037	H2-Q1; Hspa5; Tnf;
	ZBP1(DAI) mediated induction of type I IFNs	2	0.007717074	0.040401153	Myd88; Tmem173;
